# Effects of a community-based healthy heart program on increasing healthy women's physical activity: a randomized controlled trial guided by Community-based Participatory Research (CBPR)

**DOI:** 10.1186/1471-2458-7-216

**Published:** 2007-08-23

**Authors:** Raha Pazoki, Iraj Nabipour, Nasrin Seyednezami, Seyed Reza Imami

**Affiliations:** 1 Department of Healthy Heart, The Persian Gulf Health Research Center, Bushehr University of Medical Science, Bushehr, I.R. Iran

## Abstract

**Background:**

Cardiovascular disease remains the leading killer of women in most developed areas of the world. Rates of physical inactivity and poor nutrition, which are two of the most important modifiable risk factors for cardiovascular disease in women, are substantial. This study sought to examine the effectiveness of a community-based lifestyle-modification program on increasing women's physical activity in a randomized trial guided by community-based participatory research (CBPR) methods.

**Methods:**

A total of 335 healthy, 25–64 years old women who had been selected by a multiple-stage stratified cluster random sampling method in Bushehr Port/I.R. Iran, were randomized into control and intervention groups. The intervention group completed an 8-week lifestyle modification program for increasing their physical activity, based on a revised form of Choose to Move program; an American Heart Association Physical Activity Program for Women. Audio-taped activity instructions with music and practical usage of the educational package were given to the intervention group in weekly home-visits by 53 volunteers from local non-governmental and community-based organizations.

**Results:**

Among the participants, the percentage who reported being active (at lease 30 minutes of moderate intensity physical activity for at least 5 days a week, or at least 20 minutes of vigorous physical activity for at least three days a week) increased from 3% and 2.7% at baseline to 13.4% and 3% (p < 0.0001) at the ending of the program in the intervention and control groups, respectively. The participants in the intervention group reported more minutes of physical activity per week (mean = 139.81, SE = 23.35) than women in the control group (mean = 40.14, SE = 12.65) at week 8 (p < 0.0001). The intervention group subjects exhibited a significantly greater decrease in systolic blood pressure (-10.0 mmHg) than the control group women (+2.0. mmHg). The mean ranks for posttest healthy heart knowledge in the intervention and control groups were 198.91 and 135.77, respectively (P < 0.0001).

**Conclusion:**

An intervention based on CBPR methods can be effective for the short-term adoption of physical activity behavior among women. The development of participatory process to support the adequate delivery of lifestyle-modification programs is feasible and an effective healthcare delivery strategy for cardiovascular community health promotion.

**Trial Registration:**

ACTRNO12606000521527

## Background

Cardiovascular disease (CVD) remains the leading killer of women in most developed areas of the world. Coronary heart disease (CHD) accounts for the majority of CVD deaths in women. Because CHD is fatal, and because nearly two thirds of women who die suddenly have no previously recognized symptoms, it is essential to prevent CHD [1].

It is estimated that a woman in the United States dies of CVD every minute [2].

Public awareness of the scope of CVD and death in women is still lacking. In a recent study, only 46% of women knew that CVD was the leading cause of death in women [3].

Essentially, all CVD risk factors and the strategies for preventing them among men are also important for women. This issue, however, requires a gender lens, because the magnitude of risk factor effects may be different in men and women [4].

Lack of physical activity has been shown to be a strong independent risk factor for death from coronary heart disease. In a meta-analysis, Berlin and Coditz [5] calculated a 1.9 fold increased relative risk for CHD mortality associated with a sedentary lifestyle compared with a vigorously active lifestyle. Data also indicate that physical activity, including moderate-intensity exercise such as walking, is associated with substantial reduction in risk of total and ischemic stroke in a dose-response manner [6]. Cross-sectional studies of mid-life and older women have found associations between physical activity and a number of health-related variables, including blood pressure, high-density lipoprotein cholesterol, obesity, anxiety, and depression [7-10]. Rates of physical inactivity and poor nutrition, which are two of the most important modifiable risk factors for CVD in women, are substantial [11], but interventional studies for reducing theses modifiable risk factors in women have been limited and often confined to experimental-settings. An experimental-setting program seems unfeasible for the general public, since lifestyle modification should be continued for a long-term. On the other hand, a community-based program is generally acceptable and feasible for all participants. Unfortunately, there are very few community-based lifestyle-modification programs on cardiovascular risk factors including physical activity in women [12-14]. To address the epidemic of physical inactivity, obesity, and poor dietary practices among American women, the American Heart Association as part of its National Women's Heart Disease and Stroke Campaign, launched the Choose to Move program, a 12-week, self-help lifestyle intervention Program [11].

The public health burden of a sedentary lifestyle emphasizes the need for interventions that can reach enough women to make a health impact on a broad, public health scale [14]. Community-based participatory research (CBPR) has been identified as a key strategy in effectively reducing health disparities in underserved communities [15,16]. CBPR is a collaborative, partnership approach to research that equitably involves, for example, community members, organizational representatives, and researchers in all aspects of the research process. Partners contribute their expertise and share responsibilities and ownership to increase understanding of a given phenomenon, and incorporate the knowledge gained with action to enhance the health and well-being of community members [17].

There are two studies which used CBPR to impact community health promotion in cardiovascular risk factors [18,19]. We conducted a randomized controlled trial, a community-based and community-driven intervention, in which women were randomly assigned to the intervention and age-matched control groups. The purpose of this study was to explore the feasibility and effectiveness of using CBPR approach for physical activity promotion in women. To the best of our knowledge, this study is the first to examine the effectiveness of a community-based lifestyle-modification program on increasing women's physical activity in a randomized controlled trial guided by CBPR methods.

## Methods

### Engaging the community

Twelve group sessions were conducted to find out priorities in health research in Bushehr Port. The participants were community members, academic researchers, health care providers, and policy-makers. Non-communicable diseases including cardiovascular diseases were the major concerns of the groups. The result of this need assessment, the incidence of myocardial infarction in women in Bushehr (156.61 per 100,000), and data about the prevalence of coronary artery disease risk factors were presented to members of Bushehr Province Women Commission and representatives of 26 non-governmental organizations in a meeting. They convinced the importance of intervention programs for prevention of cardiovascular diseases in Bushehr.

### Partnerships

We formed a community advisory board consisting of some members of Bushehr Province Women Commission, local non-governmental organizations (NGOs), and representatives of three community-based organizations (CBOs). The advisory board was instrumental in tailoring the study to the target community. For example, they suggested that local health volunteers also be referred to as health promoters. The advisory board also reviewed training program material, reduced the duration of intervention from 12 weeks to 8 weeks, and offered insight into the recruitment of trainers. Cultural-appropriateness, readability, and comprehension of the educational material were discussed in the advisory board.

The recommendations of the advisory board were shared by health-related CBOs and academic researchers in Bushehr University of Medical Science. Publicity concerning the study appeared in the local newspapers and on TV.

### Training volunteer trainers

We used train-the-trainer approach, as a mandatory component of the program. Thus, a healthy heart trainer group consisting of 53 volunteers from local health volunteers, the members of the Persian Gulf Women Organization (as an NGO), and two CBOs were recruited. The healthy heart trainer group (HHTG) was not given a salary. They were offered four healthy heart workshops by the staffs of the Persian Gulf Health Research Center in collaboration with Bushehr Province Health Affairs.

The Persian Gulf Health Research Center and the community advisory board developed the educational curriculum. The training activities involved the development of participatory process and capacity building to support the adequate delivery of the interventions. Each member of HHTG was responsible for training five women in the intervention group. HHTG was supervised by six community members of the advisory board (as leaders), so that the advisory board would regularly stay in contact with the group. These leaders and HHTG met on a weekly basis to deepen their involvement in the intervention process. The researchers were also in close contact with the leaders.

### Community Sampling

In phase I of the study, a multiple-stage stratified cluster random sampling technique was used to select women aged 25–65 years from Bushehr Port. The estimated sample size (E/S = 0.4, two-sided alpha = 0.01 & beta = 0.10) per group (intervention & control) was 188 subjects. The selected women were informed about the study through a letter given door-to-door by the survey groups. After a primary education about cardiovascular disease and its associated risk factors, they were invited to participate in the screening program in a 12–14 fasting state in the following morning to the Persian Gulf Health Research Center.

The screening program, as presented below, was consisted for diabetes, hyperlipidemia, and ischemic heart disease. Those women who had not history or evidence of angina pectoris, myocardial infarction, cerebral stroke, renal disease, severe arthritis, lung disease, or drug consumption were randomized into control and intervention groups.

### Intervention

The intervention program was designed to permit women to easily incorporate physical activity and healthy eating changes into their lifestyle.

The subjects of interventional group were invited to the Persian Gulf Health Research Center; a detailed program material and four easy-to-read booklets consisting material about cardiovascular diseases, risk factors of coronary artery disease, smoking and nutrition for healthy heart were given to them.

A program for increasing physical activity (Exercise for Healthy Heart, EHH) was designed to teach women how to incorporate a daily routine of physical activity into their lives in creative and practical ways, based on Choose to Move(CTM) program; an American Heart Association Physical Activity Program for Women [11]. Choose to Move was developed to help women in the contemplative and preparatory stages of physical activity move to the next stage. Participants are asked to begin with 10 minutes per day of moderate-intensity physical activity; women are encouraged to do 30 minutes of physical activity daily. The 8-week EHH was a fully revised form of Choose to Move program.

EHH booklets including audio-taped activity instructions with music and practical usage of the educational package were given to the intervention group by members of HHTG. Each trainer covered five subjects in the intervention group. The members of HHTG met the participants in a weekly schedule. They delivered EHH educational packages in their face-to-face weekly home-visits. Each participant had a total of eight 1.5-hour face-to-face educational interview sessions with her trainer. As previously mentioned,. the trainers were community members who were offered four workshops for training in healthy lifestyle including the package of EHH.

### Measurements

On arrival at the survey site, information on their age, sex, marital status, education, smoking, estrogen, and drugs for angina, hypertension, diabetes, and dyslipidemia were recorded using WHO MONICA questionnaire [20] by trained interviewers. To evaluate physical activity behavior at both registration time and week 8, participants complete a 7-Day physical activity recall questionnaire based on the BRFSS; USA/CDC, 2002) and the Countrywide Integrated Non-communicable Diseases Intervention (CINID) program questionnaire [20,21]. Participants were classified as active at the recommended level if they reported sufficient physical activities of moderate intensity (i.e., > or =30 minutes per day, > or =5 days per week) or of vigorous intensity (i.e., > or =20 minutes per day, > or =3 days per week) [22]. Blood pressure was assessed twice at the right arm after a 15-min rest in the sitting position, using a standard mercury sphygmomanometer. Height and weight were measured using a stadiometer. Heavy outer garments and shoes were removed before measuring height and weight. Body Mass Index (BMI) was calculated. Waist circumference was defined at the midway level between the costal margins and the iliac crests. Hip circumference was measured at the level of the greater trochanters. A resting 12-lead electrocardiogram was performed.

A fasting blood sample was taken, all samples were promptly centrifuged, separated and analyses were carried out at the Persian Gulf Health Research Center on the day of blood collection using a Selectra 2 auto analyzer (Vital Scientific, Spankeren, The Netherlands). Glucose was assayed by enzymatic (glucose oxidize) colorimetric method using a commercial kit (Pars Azmun Inc; Tehran, Iran). Serum total cholesterol was measured using a cholesterol oxidase phenol aminoantipyrine and triglycerides using a glycerol-3 phosphate oxidize phenol aminoantipyrine enzymatic method.

The research team developed a questionnaire to identify the participants' healthy heart knowledge and awareness. The 49-item questionnaire was consisted of 29 nutrition items, 10 physical activity items, 8 smoking items, and 2 general items regarding healthy heart.

### Ethics & Statistical analysis

All of the subjects received detailed information regarding the purpose and nature of the study, and gave their informed consent before enrollment. This study was approved by the Ethics Committee in Bushehr University of Medical Sciences.

We used x2 tests to compare differences in proportions, unpaired t tests for continuous normally distributed variables, and Mann-Whitney tests for normally distributed variables (healthy heart knowledge score).

A value of P < 0.05 was established as statistically significant. Statistical analysis was performed with an IBM computer using the SPSS 10 statistical software package (SPSS Inc., Chicago, IL).

## Results

Of the 589 women surveyed, 109 subjects who had history or evidence of angina pectoris, myocardial infarction, cerebral stroke, renal disease, severe arthritis, lung disease, or drug consumption were excluded. Of 480 women who remained, 385 subjects agreed to participate in the trial. They were randomly entered in the intervention group (179 cases) or the control group (179 cases). A total of 170 subjects in the intervention group completed 8-week program. They were compared with 165 age-matched women in the control group who returned for the required measurements in the second session. The subjects ranged in age from 25 to 64 years (mean age, 39.4 years).

There were no significant differences in baseline demographic characteristics of the intervention and control groups; including socioeconomic status data.

The characteristics of the two groups at baseline and after 8-week program were summarized in Table 1. At baseline, there was no significant body mass Index (BMI), waist to hip ratio, systolic and diastolic blood pressures, fasting blood sugar, lipid profile and physical activity levels differences between the study groups.

**Table 1:** Physical and clinical characteristics and healthy heart knowledge of the subjects before and after 8-week program for intervention and control groups

	Intervention		control		
	Before	After	Before	After	P value

Body Mass Index(kg/m^2^)	28.02(4.74)§	27.53(4.49)	27.82(5.39)	28.05(8.42)	NS
waist to hip ratio (cm)	0.91(0.06)	0.87(0.07)	0.92(0.07)	0.88(0.07)	NS
Systolic blood pressure (mmhg)	111.28(14.47)	110.28(17.17)	111.38(13.40)	113.60(12.64)	0.04
Diastolic blood pressure(mmhg)	68.97(12.11)	72.62(9.95)	69.95(11.26)	74.19(8.87)	NS
Total cholesterol(mg/dl)	193.15(37.94)	196.88(40.38)	199.15(40.14)	200.82(42.18)	NS
Triglyceride (mg/dl)	127.66(74.84)	133.38(73.94)	130.23(69.11)	140.21(73.77)	NS
Fasting Blood Sugar (mg/dl)	81.94(14.46)	86.65(12.58)	83.51(18.80)	87.54(14.77)	NS
Knowledge Score (out of 49)		41.12 (0.26)		38.01(0.39)	<0.0001
Moderate physical activity (minutes/week)	17.59(5.23)	116.46 (18.03)	26.59 (11.61)	37.15 (12.42)	0.03
Vigorous physical activity (minutes/week)	4.09 (1.81)	21.38 (8.70)	3.61 (1.65)	3.02(1.03)	0.001

§Values are mean (SD), except for physical activity and knowledge score which are mean (SE)

Among the participants, the percentage of those who reported being active increased from 3.0% and 2.5% at baseline to 13.4% and 3.0% (p < 0.0001) at the program end in the intervention and control groups, respectively. There were statistically significant differences between the two groups in regard to changes in moderate (p = 0.03) and vigorous (p = 0.001) physical activities (Table 1). We found that participants in the intervention group reported more minutes of physical activity per week (mean = 139.81, SE = 23.35) than women in the control group (mean = 40.14, SE = 12.65) at week 8 (p < 0.0001).

As shown in Table 1, there were no significant differences between the groups with regard to BMI, WHR, serum sugar and lipid levels, and diastolic blood pressure changes from baseline. However, the intervention group subjects exhibited a significantly greater decrease in systolic blood pressure (-10.0 mmHg) than the control group women (+2.0 mmHg).

Total heart-healthy awareness and knowledge score at posttest was 6.26% higher in the intervention than in control subjects.

The mean ranks for posttest healthy heart knowledge in the intervention and control groups were 198.91 and 135.77, respectively (U = 8736.5 P < 0.0001).

## Discussion

Results indicated that participants in the intervention group spent significantly more minutes of physical activity (of at least moderate intensity) comparing to participants of the control group in 8 weeks. The study demonstrated that volunteers could be successfully trained to deliver an educational program designed to increase physical activity in women. Furthermore, volunteers of the Healthy Heart Trainer Group were able to effectively recruit members of their community through the usage of their social networks, to conduct the health-promotion program.

Chose to Move (CTM) program was designed to be delivered in a self-help format, without in-person supplementation; it was mainly a mail-mediated lifestyle intervention program [11]. However, our study educational material was based on a revised CTM program but we added component of in-person contact with lay health educators. In a CBPR approach, we used community volunteers to deliver healthy heart physical activity packages to women. Our program was provided a low cost intervention, enrolled a large number of women, and had a high rate of success for those who completed it. This program produced a feasible, effective, and appropriate model for low to mid-income countries. This program also showed that a social marketing approach – promoting a targeted, self-help lifestyle intervention program, designed to increase physical activity, for healthy heart – can reach a large number of women and help them to positively change their behavior within 8 weeks.

With the shift in emphasis towards population level interventions to change health risk behaviors, community based participatory intervention programs such as the Persian Gulf EHH program have great appeal. These results compare favorably with other self monitoring interventions that are tailored to women's needs, in cardiovascular healthy lifestyles [18,19]. Participatory research methods were used to design an outreach program through a collaborative partnership between UCLA School of Nursing, Los Angeles County Department of Health Services, and members of one community of underserved Latinos; the findings of this study supported the feasibility and effectiveness of using Lay Health Advisors for cardiovascular health promotion in a low-income community [18].

The para Su Corazon (Health for Your Heart), a community-based outreach program among Latinos, established by the National Heart, Lung, and Blood Institute partnered with the National Council of La Raza, also worked well in seven pilot programs because of the successes of the community health workers and the support of the community based organizations [19].

We implemented many elements of CBPR in EHH program, with community members engaged early in the process and throughout the project. We formed a community advisory board consisting of some members of Bushehr Province Women Commission, local NGOs, and representatives of three CBOs. Through meetings, and frequent subcommittee meetings, the university researchers and the members of community began to understand and respect each other's expertise and respective. The study team relied on input from members of the community to guide the nature and structure of the intervention. The rationale for pursuing community participation includes promoting positive health behavioral change; improving service delivery; mobilizing human, financial and other material(including in-kind) resources for health services; and as a means of empowering the community [23]. CBPR motivates co-learning and sharing expertise by researchers and community members [17]. It differed from more traditional community-based research, which favors work conducted in a community setting, but with limited community involvement [24].

The employment of a CBPR approach in our research enhanced the design, conduct, and conclusions of our study. Our inclusion of program partners in the interpretation of study findings led to more dynamic modes of analysis and more reflective conclusion drawing processes that also proved useful for program and community development.

In summary, the current findings support the use of community-based approach as a feasible and effective healthcare delivery strategy for community health promotion at a grassroots level, and as having promising indicators of sustainability over time. Sustainability is an important issue for community-based health-promotion interventions to make a difference over time. Some studies have suggested correlates of sustainability in terms of intervention characteristics, such as interventions that use no paid staff [25,26].

The EHH team including the community members were not paid a salary. However, it is too soon to determine whether we have been successful in creating a sustainable cadre of community-based expert trainers for healthy heart.

There were a number of strengths of this study. The first published evaluation of a CTM program was a quasi-experimental, non-randomized study of the 1999 version [11]. Survey results indicated women increased their activity, but there was high attrition and a lack of comparison groups [11]. For the first time, Napolitano et al [14] designed a controlled trial which compared CTM print-based program to another print-based physical activity promotion program (i.e., Jumpstart) and a Wellness contact control group. Our study was the second one which examined the efficacy of the American Heart Association's Choose to Move (CTM) program for physical activity promotion delivered via in-person home visits in comparison to a control group. Napolitano et al [14] and our study provided the first controlled comparison of AHA materials which may provide information for the AHA in the design and dissemination of future programs.

The EHH program participants were free of cardiovascular disease who selected in general population, irrespective to their baseline cardiovascular risk factors. Individuals who already have one or more mild cardiovascular risk factors still could be good candidates for a community-based participatory lifestyle-modification program. A community-based lifestyle-modification program that consisted of mild aerobic exercise and a mild hypo caloric diet was considered to be practically effective for reducing multiple cardiovascular risk factors [13].

For feasibility, our study used self-reported data for physical activity behavior, a limitation for validating results. A prospective study by Blair et al, however, found that self-reported physical activity was the predominant predictive factor of cardio respiratory fitness among adults in all age and sex subgroups they analyzed [27]. In addition, the questions that measured physical activity have been field tested and are used in the BRFSS; USA/CDC, 2002) and the Countrywide Integrated Non-communicable Diseases Intervention (CINID) program [20,21]. Future studies should include some measure of fitness of women in the community, such as a 2-km walking test or the Rockport Walking Test for field settings [28].

In two American studies, high HDL-C levels in women have been shown to be important in providing a protective effect from coronary heart disease [29]. Several cohort studies found that increases in physical activity were associated with favorable changes in HDL-C [30,31]. However, exercise intervention studies have not demonstrated consistently an improvement in women's lipid and lipoprotein profiles [32]. In EHH program, no statistically significant decrease in lipid levels corresponding to a change in physical activity was found; it may reflect the lack of sufficient change in exercise behavior or limited power of the study to detect small effects in short terms. Total of awareness, knowledge, and perceptions about heart disease, and healthy heart lifestyle was 6.26% correct higher in the intervention than in the control group. It seems that EHH program succeeded at improving heart-health awareness and creating a cultural environment for learning heart-health information to promote changes in lifestyle behaviors among women of the intervention group.

In this work, we found that CBPR is an important research approach in addressing cardiovascular prevention among women. It is compatible with cultural values. Continued effort needs to be directed towards creating systems and structures to support researchers in utilizing this method.

In conclusion, it is possible to deliver heart-healthy program through existing community infrastructures. This program provides an important model for public health, voluntary, and other health organizations of population-based, targeted low cost self-help programs that support objectives for physical activity and cardiovascular health.

## Conclusion

An intervention based on CBPR methods can be effective for the short-term adoption of physical activity behavior among women. The development of participatory process to support the adequate delivery of lifestyle-modification programs is feasible and an effective healthcare delivery strategy for cardiovascular community health promotion.

## Competing interests

The author(s) declare that they have no competing interests.

## Authors' contributions

RP and IN had conceived and designed the study as well as wrote the manuscript; NS and SRI assisted in the field survey. All authors read and approved the final manuscript.

## Pre-publication history

The pre-publication history for this paper can be accessed here:


